# Supplementary material for: Artificial fingerprints engraved through block-copolymers as nanoscale physical unclonable functions for authentication and identification
Source: Nat Commun. 2024 Dec 11;15:10576. doi: 10.1038/s41467-024-54492-8 (PMC11634899; doi:10.1038/s41467-024-54492-8)
Supplement: Supplementary file 1 — Supplementary Information [file 41467_2024_54492_MOESM1_ESM.pdf]

Supplementary Information – Artificial fingerprints engraved through block-copolymers as nanoscale physical unclonable functions for authentication and identification

Irdi Murataj[1], Chiara Magosso[1,2], Stefano Carignano[3], Matteo Fretto[1], Federico Ferrarese Lupi[1*], Gianluca Milano[1*]

[1]Advanced Materials Metrology and Life Sciences Division, INRiM (Istituto Nazionale di Ricerca Metrologica), 10135 Turin, Italy.

[2]Department of Electronics and Telecommunications, Politecnico di Torino, 10129 Turin, Italy.

[3]Barcelona Supercomputing Center, 08034, Barcelona, Spain.

These authors contributed equally to this work: Irdi Murataj, Chiara Magosso

*e-mails: f.ferrareselupi@inrim.it, g.milano@inrim.it

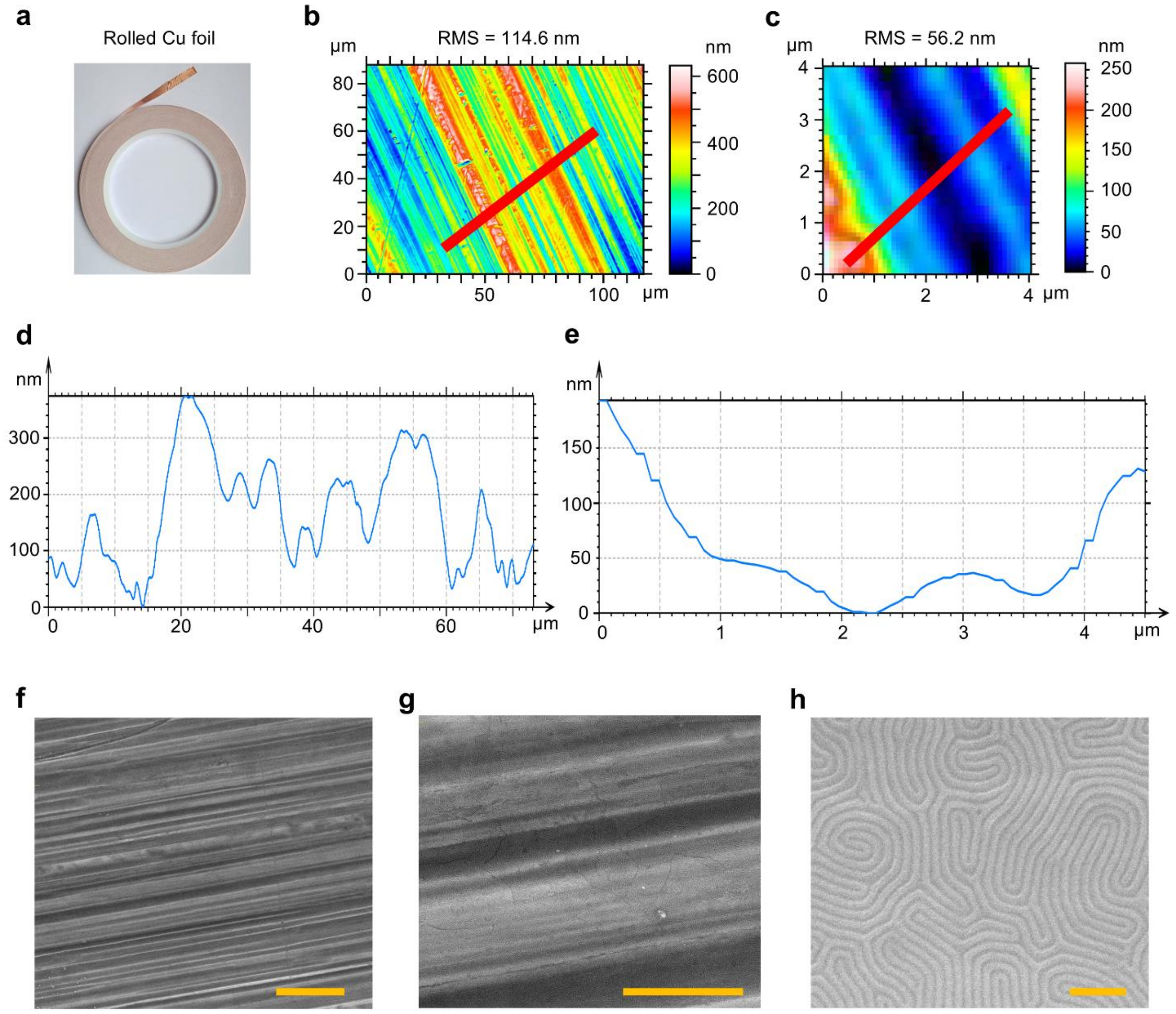

Supplementary Figure S1 | BCP self-assembly on a rolled metal foil. (a) Photograph of the Cu metal foil used as a substrate for the BCP self-assembly. (b, c) Optical profilometry topographic maps at two different scales and (d, e) corresponding height profiles traced by the red lines in (b, c), showing the high roughness of the substrate. The RMS value measured in (b) is 114.6 nm; the RMS value measured in (c) is 56.2 nm. SEM micrographs at different magnifications showing the high roughness of the substrate (f and g), and a high magnification image showing self-assembled BCP over the selected object (h). Scale bars are set to 10 μm for (f), 3 μm for (g) and 200 nm for (h).

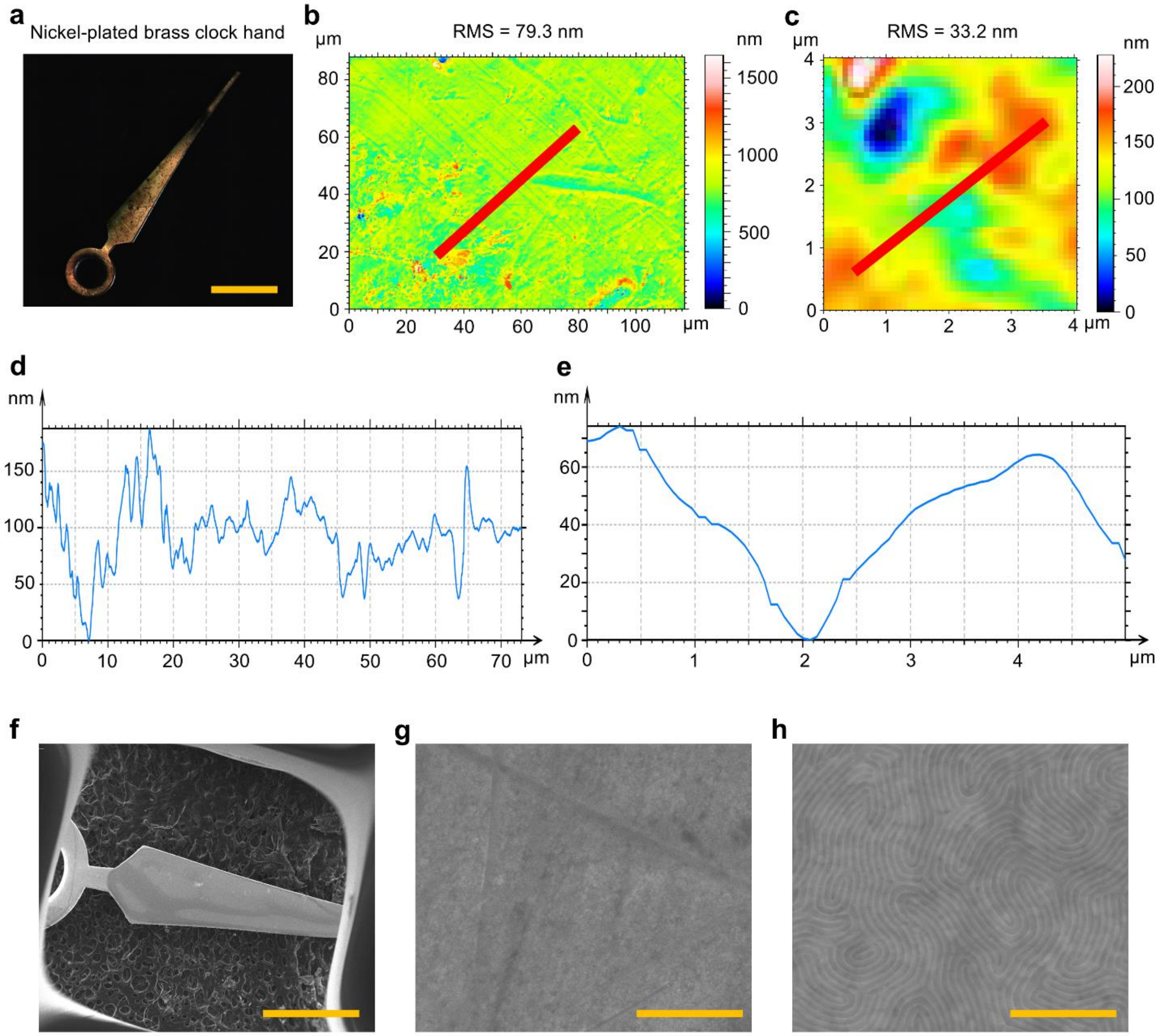

Supplementary Figure S2 | BCP self-assembly on a nickel-plated brass clock hand. (a) Optical image of the nickel-plated brass clock hand used as a substrate for the BCP self-assembly. Scale bar is set to 4 mm. (b, c) Optical profilometry topographic maps at two different scales and (d, e) corresponding height profiles traced by the red lines in (b, c), showing the high roughness of the substrate. The RMS value measured in (b) is 79.3 nm; the RMS value measured in (c) is 33.2 nm. SEM micrographs at different magnifications showing the high roughness of the substrate (f and

g), and a high magnification image showing self-assembled BCP over the selected object (h). Scale bars are set to 2 mm for (f), 2 μm for (g) and 500 nm for (h).

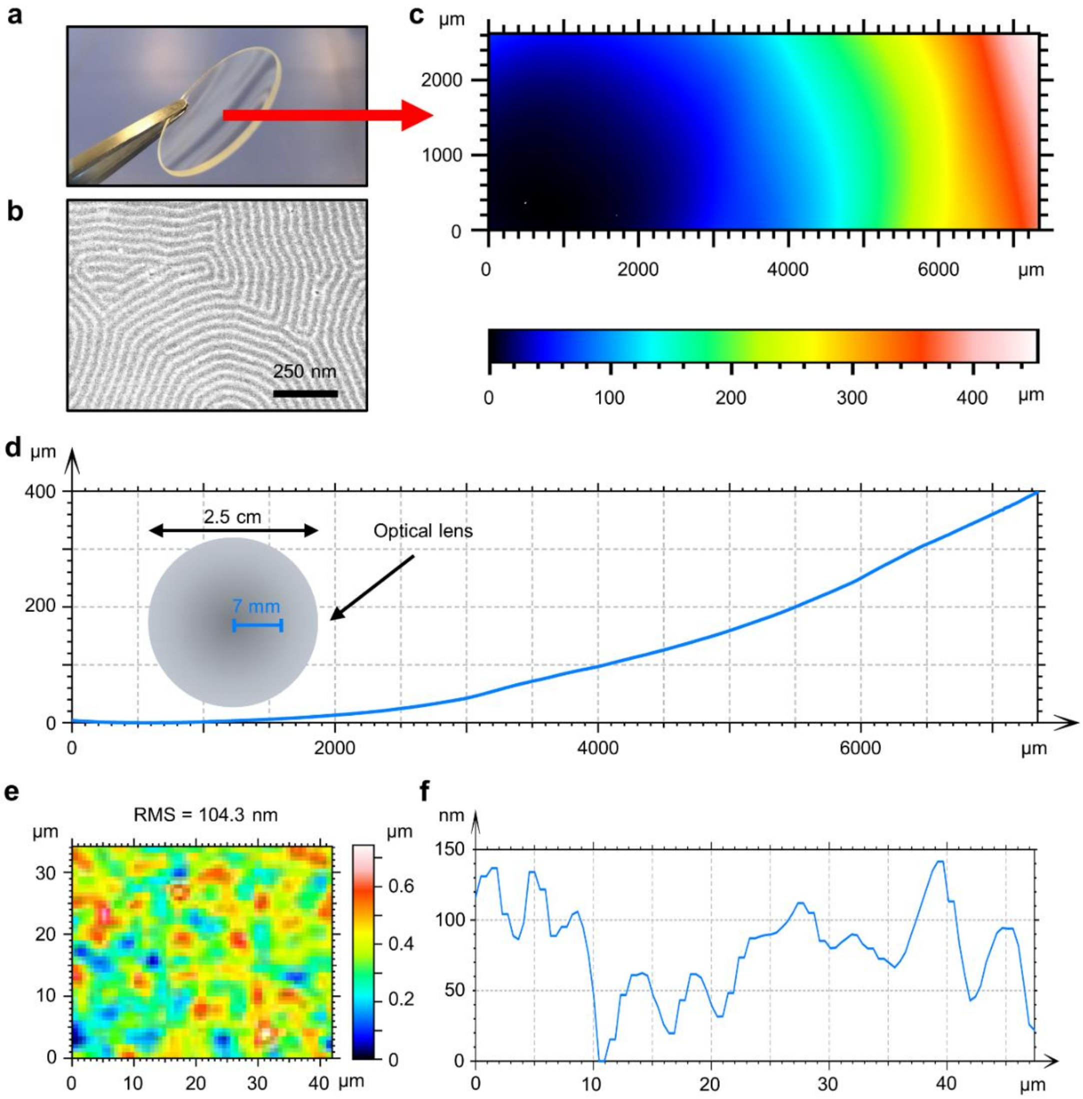

Supplementary Figure S3 | Fingerprint patterns engraved on curved and rough surface | a. Photograph of an optical glass lens and **b**. SEM image of the engraved fingerprint pattern. **c.** Optical profilometer measurement of the curvature of the optical lens on a 7.3 x 2.5 mm^2 area and **d.** relative height profile with schematic representation of the lens measured in confocal imaging mode with a 5x optical lens showing the lens curvature. **e.** Optical profilometer measurement of the roughness of the optical lens on a 42 x 35 μm^2 area with a 20x optical lens (the measured RMS value is 104.3 nm) and **f.** the relative profile traced by the red line in **e**. The RMS roughness

calculated over several 4 x 4 μm^2 regions is in the range of about 50-170 nm, depending on the specific area. In this context, the BCP self-assembly was observed to occur unregarding the specific local roughness.

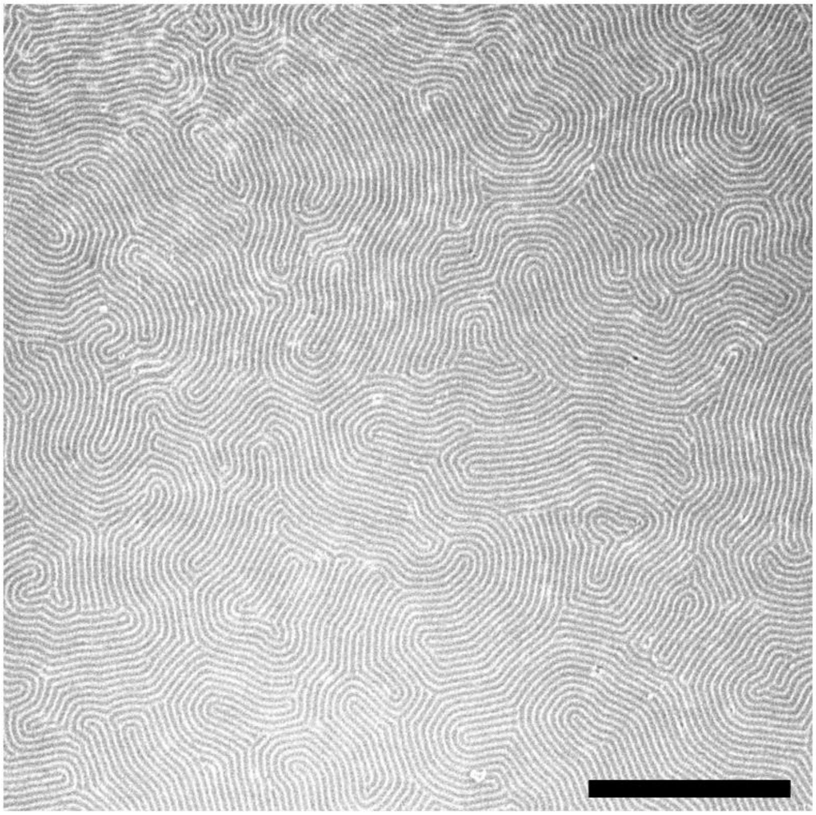

Supplementary Figure S4 | Large area SEM image of a fingerprint pattern engraved on curved and rough surface. The SEM image represents a fingerprint pattern over an area of 4 µm x 4 µm engraved on the optical lens described in Supplementary Figure S3. The scale bar is set to 1 µm.

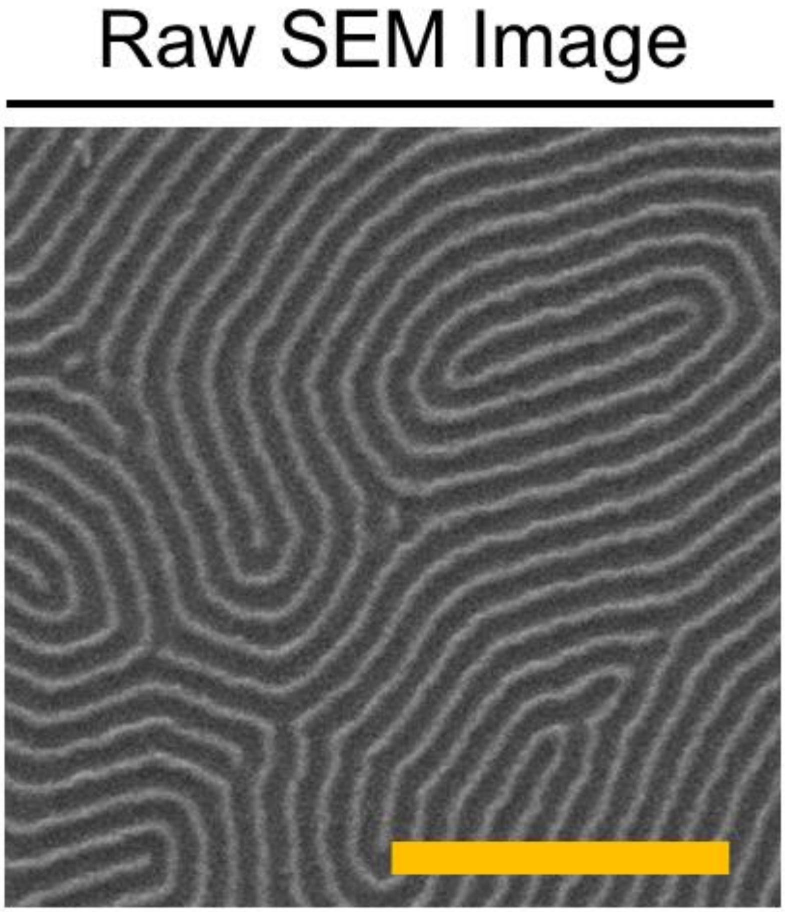

Supplementary Figure S5 | | Raw SEM image of the nanopattern. Raw SEM image exploited as example in Fig. 2c to compare conventional binarization techniques with fingerprint enhancement algorithm. Scale bar is set to 400 nm.

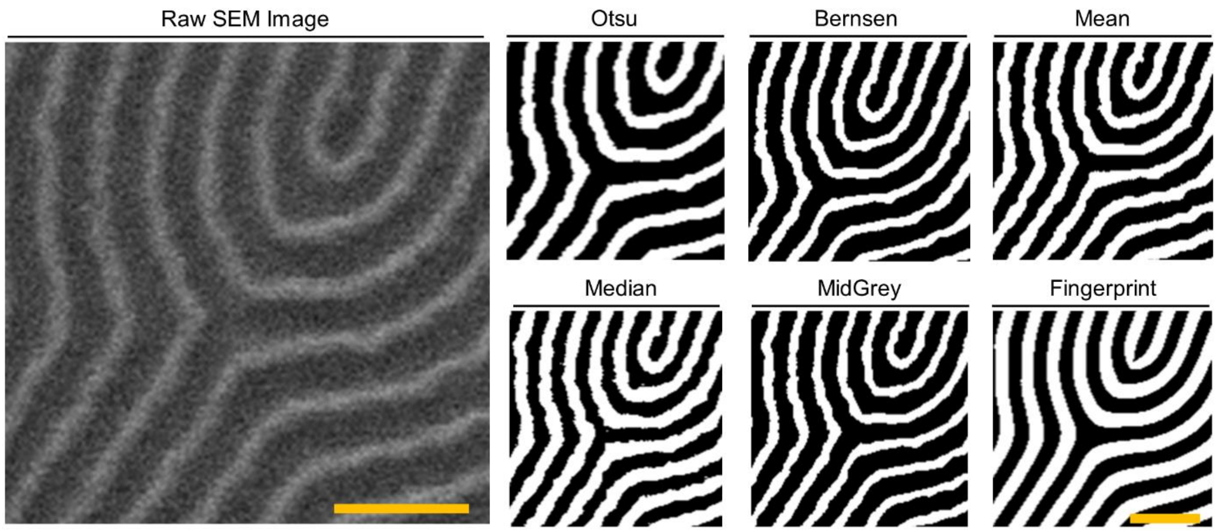

Supplementary Figure S6 | Detailed comparison of pattern extraction from different binarization techniques. Details on the comparison of conventional auto local binarization techniques with fingerprint enhancement algorithms. As can be observed, fingerprint enhancement allows obtaining a binarized image of the nanopattern with reduced noise. All scale bars are set to 100 nm.

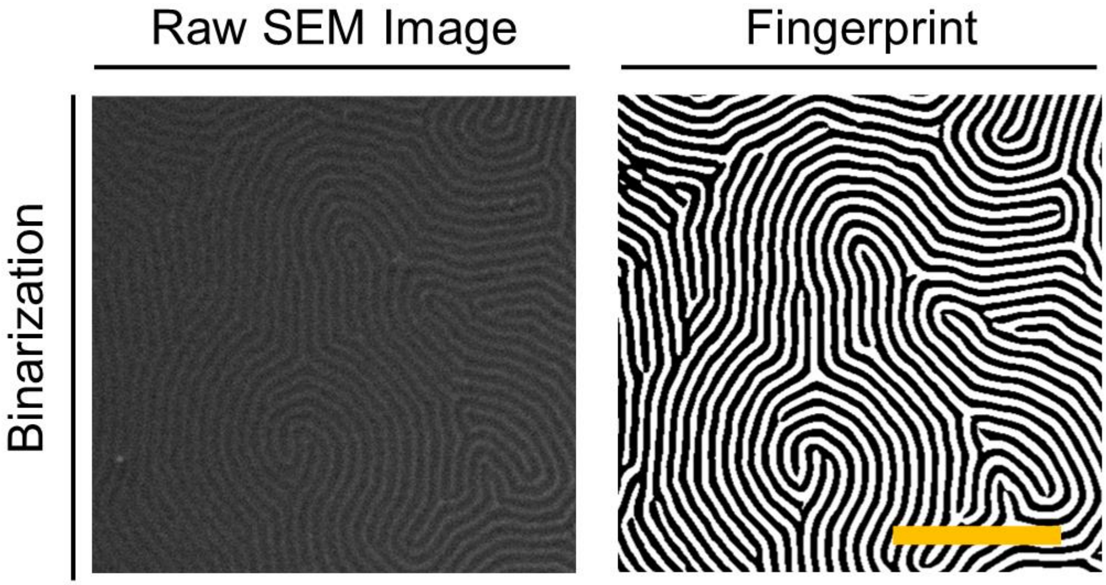

Supplementary Figure S7 | Successful binarization of low-quality SEM images by fingerprint enhancement algorithm. Example of a successful binarization of a low-quality SEM image by exploiting the fingerprint enhancement algorithm. Scale bar is set to 500 nm.

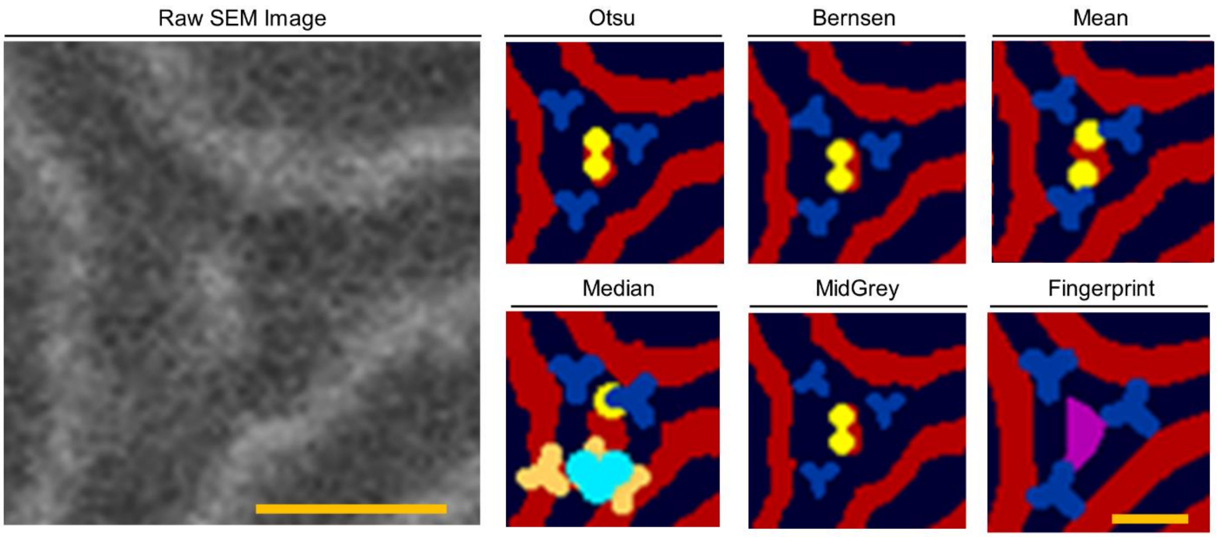

Supplementary Figure S8 | Details on the influence of the binarization technique on defect identification. Example of overestimation and improper attribution of a positive phase dot defect depicted in the raw SEM image due to improper binarization by different auto local thresholding techniques, compared to fingerprint enhancement algorithm. Otsu, Bernsen, Mean and MidGrey thresholding methods improperly attribute a positive phase dot defect to two positive terminal points. Whereas Median thresholding attributes the same defect to one positive and three negative phase terminal points along with another improper attribution to other three positive phase 3-way junctions. As can be observed, fingerprint enhancement allows obtaining a binarized image of the nanopattern with a reduced number of artifacts. All scale bars are set to 50 nm.

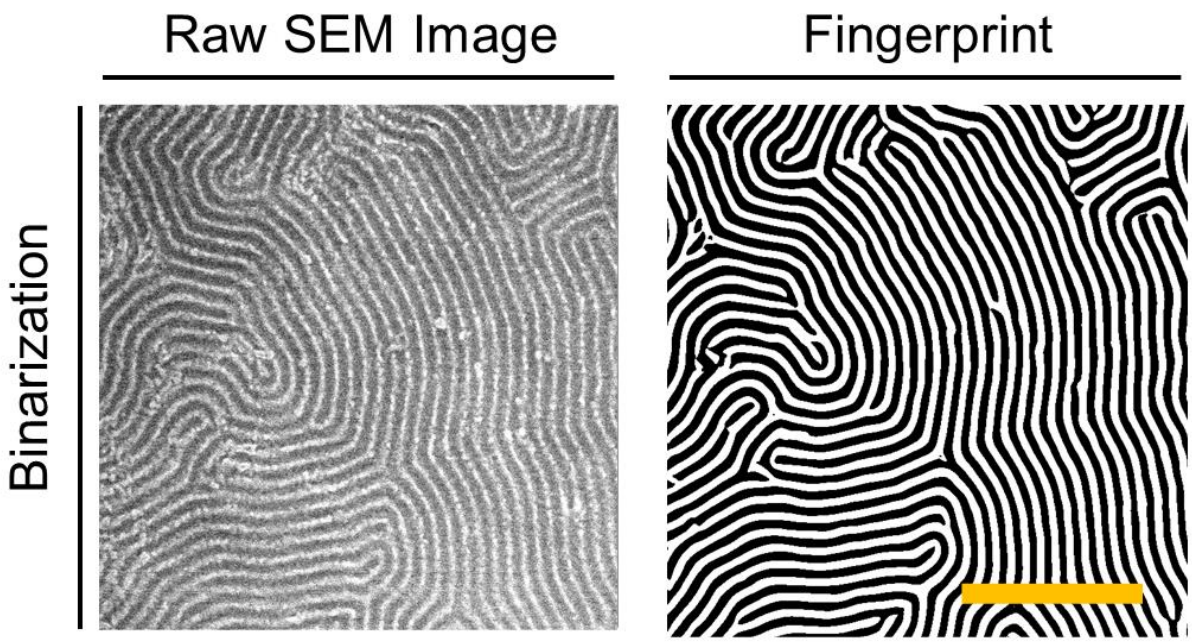

Supplementary Figure S9 | Binarization of a pattern engraved on a curved surface | Successful binarization of an SEM image of a fingerprint pattern engraved on a curved optical lens surface (refer to Supplementary Figure S3) by exploiting the fingerprint enhancement algorithm. Scale bar is set to 500 nm.

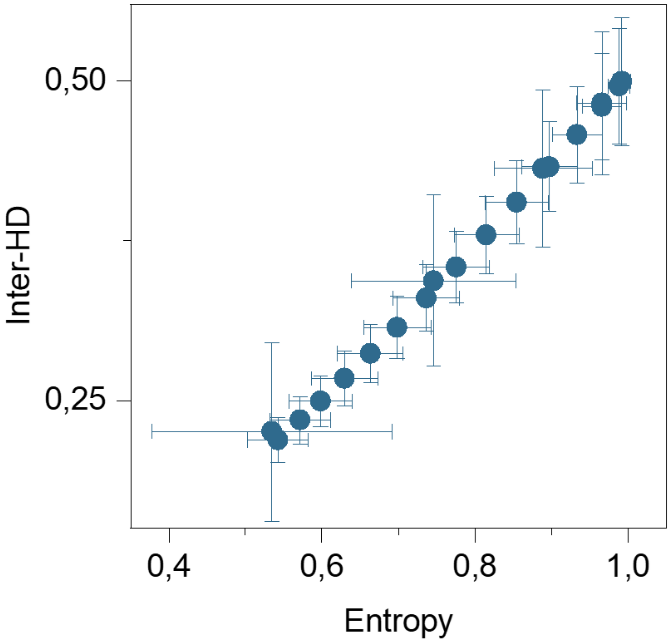

Supplementary Figure S10 | Relationship between fractional inter-HD and unit entropy of binary code matrices. This graph was obtained by considering fractional HD values and entropy at different pixel sizes during the assignment of the binary code matrix from the nanopattern (analyzed pixel sizes are the one reported in Fig. 3c, note that the entropy was not evaluated for pixel sizes of 595 and 476 nm since in this case it was observed a not null probability of having all 1-bit values across the matrix). Dots are mean values while error bars represent the standard deviation, calculated by considering 200 binary code matrices obtained from SEM images with an area of 2.38 × 2.38μm^2 acquired on different areas of the patterned sample. Note that the fractional inter-HD between different binary code matrices tends to the ideal value of 0.5 when entropy of binary code matrices tends to the ideal value of 1.

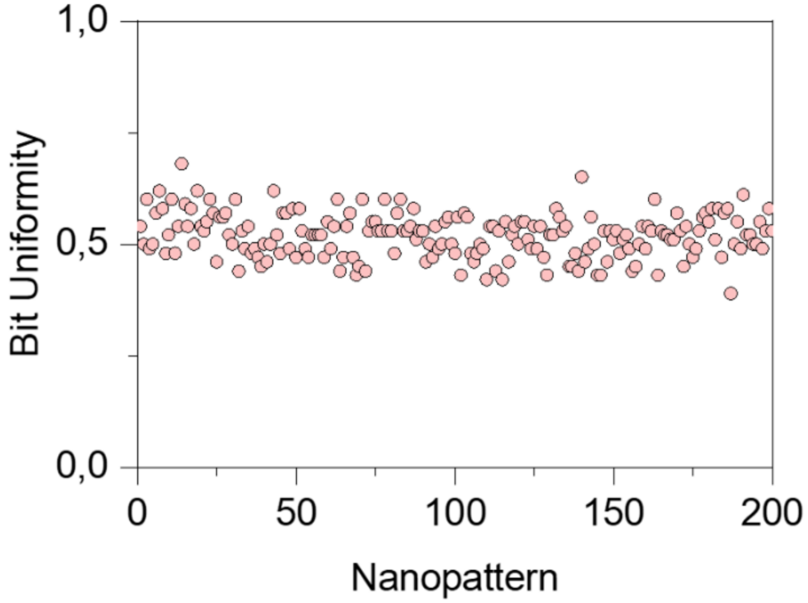

Supplementary Figure S11 | Bit uniformity of binary code matrices. The bit uniformity was evaluated on 200 SEM images of different nanopatterns, for a fixed pixel size of the binary code matrix of 238 × 238 nm^2.

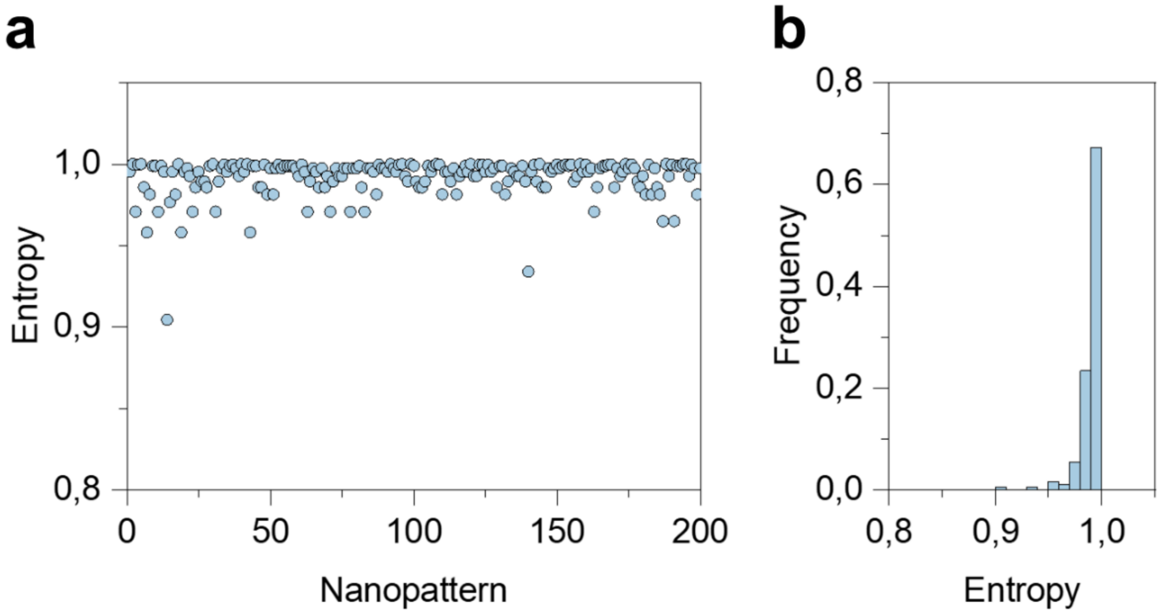

Supplementary Figure S12 | Unit entropy of binary code matrices. a. Unit entropy for 200 nanopatterns and **b.** corresponding unit entropy distribution for a fixed pixel size of the binary code matrix of 238 × 238 nm^2.

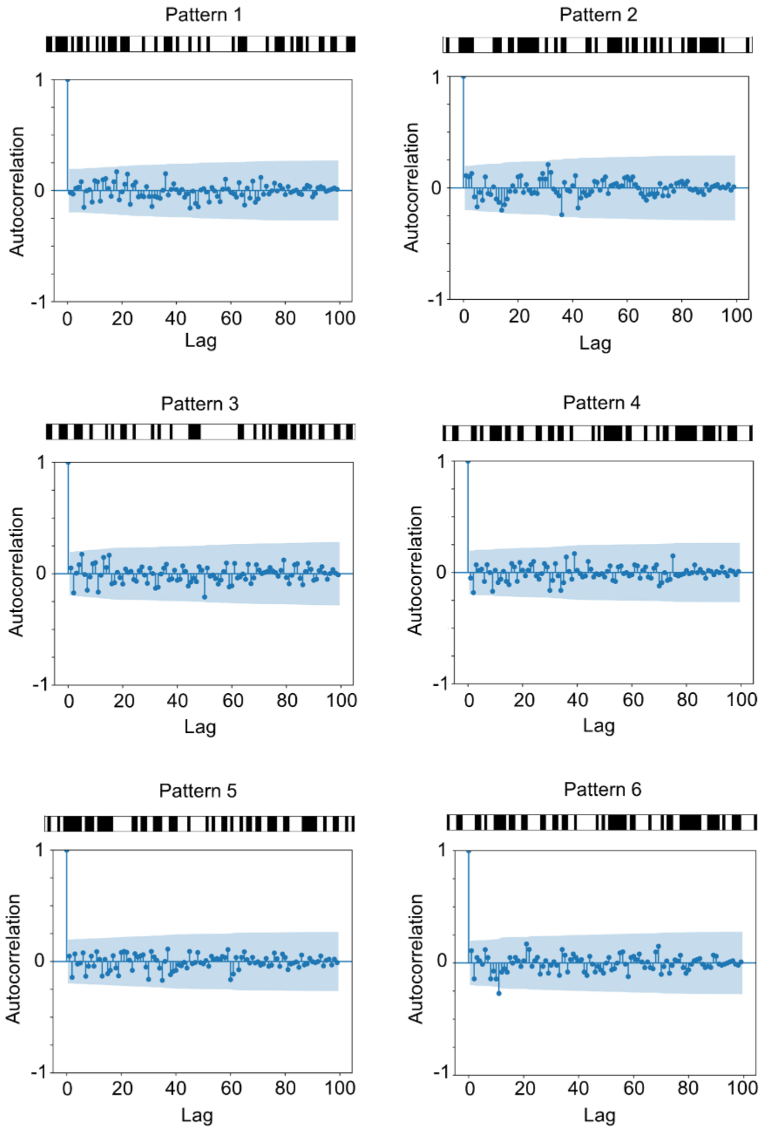

Supplementary Figure S13 | Autocorrelation function as a function of lags. Examples of autocorrelation as a function of lags for six different binary code matrices organized as an array of 100 elements by placing the rows of the matrix one after the other. As can be observed, autocorrelation values for lag > 0 mainly lay within the boundary of the 95% confidence (only few values are outside the confidence band) indicating that there is negligible correlation between pixels (note that the autocorrelation value of 1 at lag = 0 corresponds to the correlation of the pixel against itself).

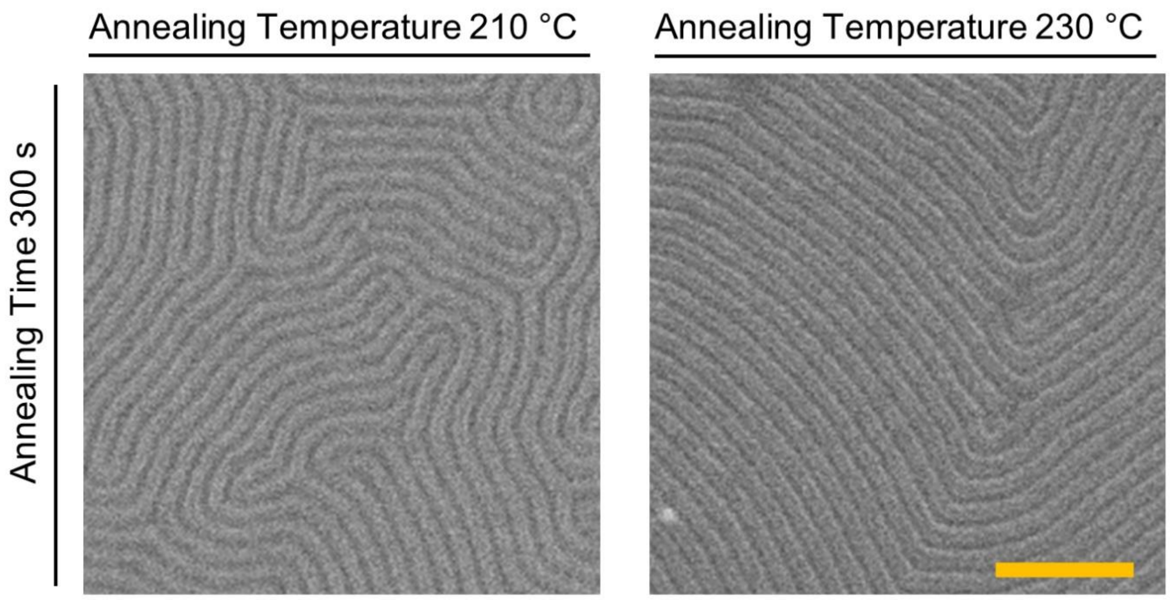

Supplementary Figure S14 | Tuning correlation length of nanopatterns depending on the self-assembly process parameters. SEM images of the lamellar structures obtained with the same polymers under different processing conditions. For a processing time of 300 s, annealing temperatures of 210 °C and 230 °C result in nanopatterns with correlation length of ~173 and ~328 nm, respectively. Scale bar is set to 250 nm.

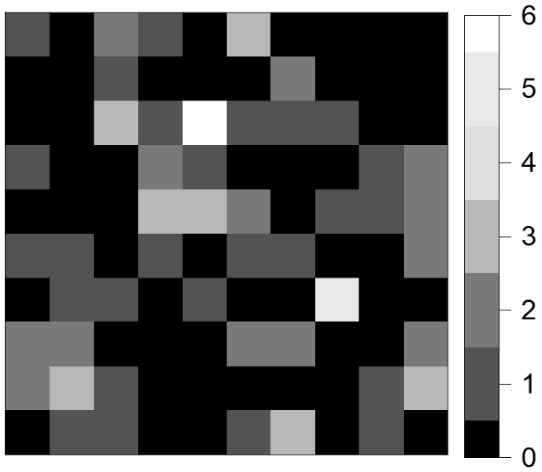

Supplementary Figure S15 | Analog version of binary code matrices based on defect density. Analog version of the binary code matrix reported in Fig. 3b, where the color intensity here represents the number of positive phase defects in the pixel area.

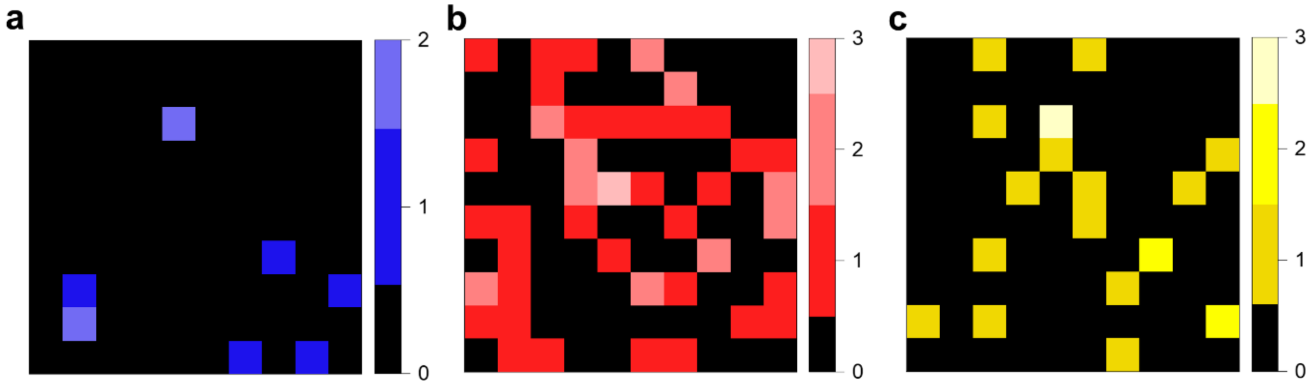

Supplementary Figure S16 | Analog version of binary code matrices based on defect types. Analog version of individual defect types of the binary code matrix reported in Fig. 3b where the color intensity represents the number of **a.** positive phase dot defects, **b.** positive phase terminal point defects and **c.** positive phase 3-way junction defects in the corresponding pixel area.

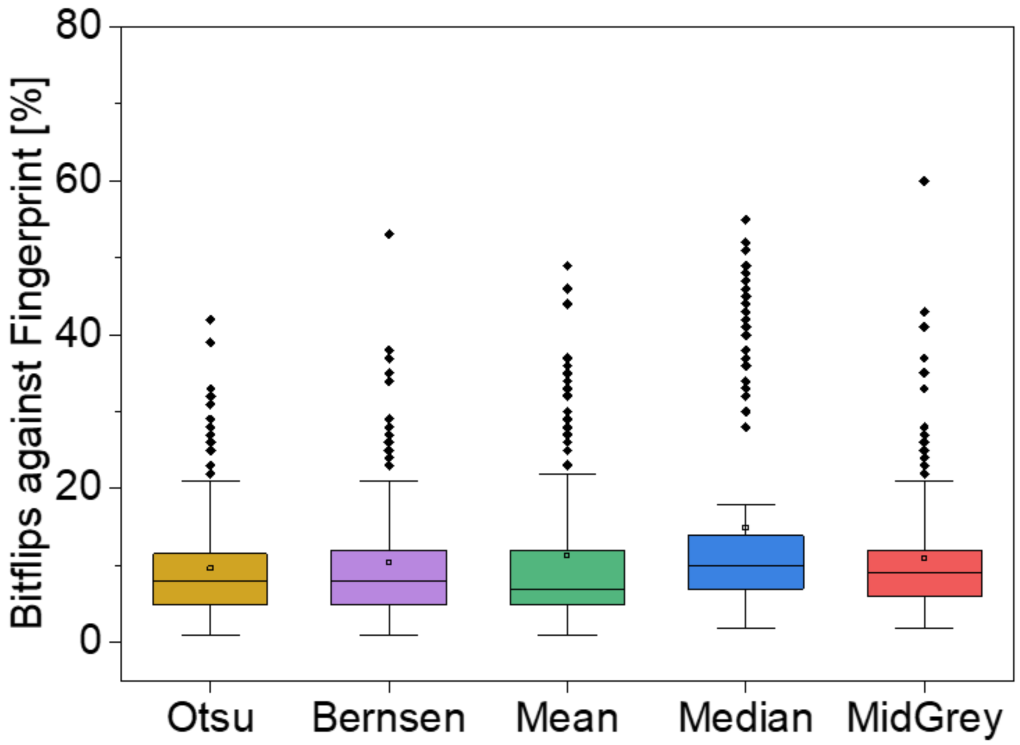

Supplementary Figure S17 | Artefact bit flips of different thresholding methods with respect to fingerprint. Box plots representing the bit flips of binary code matrices from different thresholding methods against binarization from the fingerprint enhancement algorithm. Midline represents median value, boxes the 25th and 75th percentiles, whiskers the 1.5 IQR (interquartile range), and black squares the outliers.

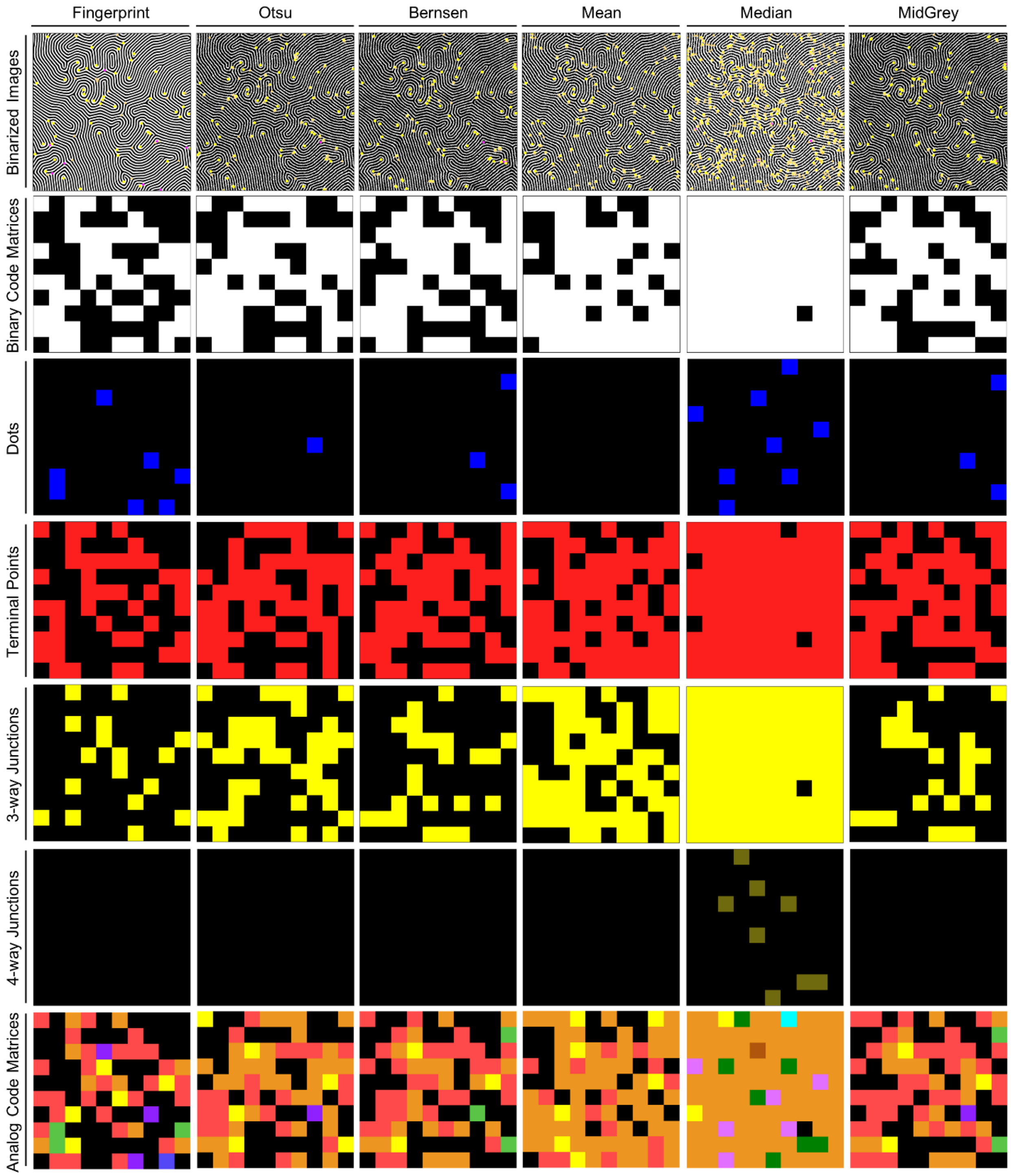

Supplementary Figure S18 | Binary code matrices comparison. Direct comparison of analog binary code matrices generated from different binarization thresholding methods of the same SEM image. The pixel is blue in presence of positive phase dot defects; pixel is red in presence of

positive phase terminal point defects; pixel is yellow in presence of positive phase 3-way junction defects; pixel is olive green in presence of positive phase 4-way junction defects; pixel is light green in presence of positive phase dot and positive phase terminal point defects; pixel is orange in presence of positive phase terminal point and positive phase 3-way junction defects; the pixel is light blue in presence of positive phase dot defects and positive phase 3-way junction defects; the pixel is dark green in presence of positive phase 3-way junction defects and positive phase 4-way junction defects; pixel is violet in presence of positive phase dot defects, positive phase terminal point and positive phase 3-way junction defects; pixel is brown in presence of positive phase dots, positive phase 3-way junction and positive phase 4-way junction defects.

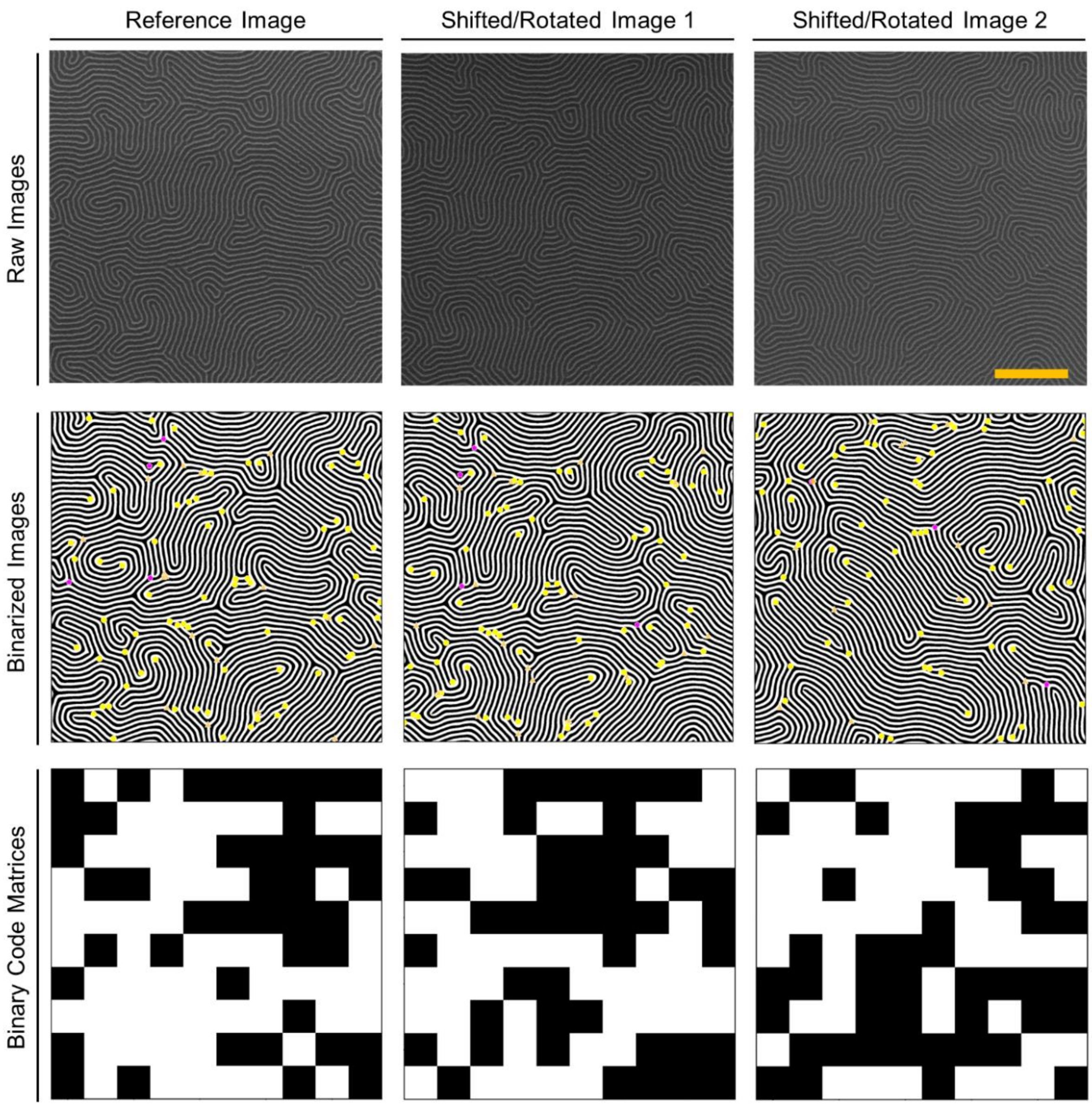

Supplementary Figure S19 | Bit flips in slightly shifted/rotated images of the same pattern. Raw images, corresponding binarized images through fingerprint enhancement and corresponding binary code matrices. As can be observed, even slight shifts and rotations of different images of the same pattern can result in strongly different binary code matrices. In this case, slightly shifted/rotated image 1 and 2 result in 47% and 55% of bit flips in the corresponding binary matrix with respect to the binary code matrix obtained from the reference image. Scale bars are set to 500 nm.

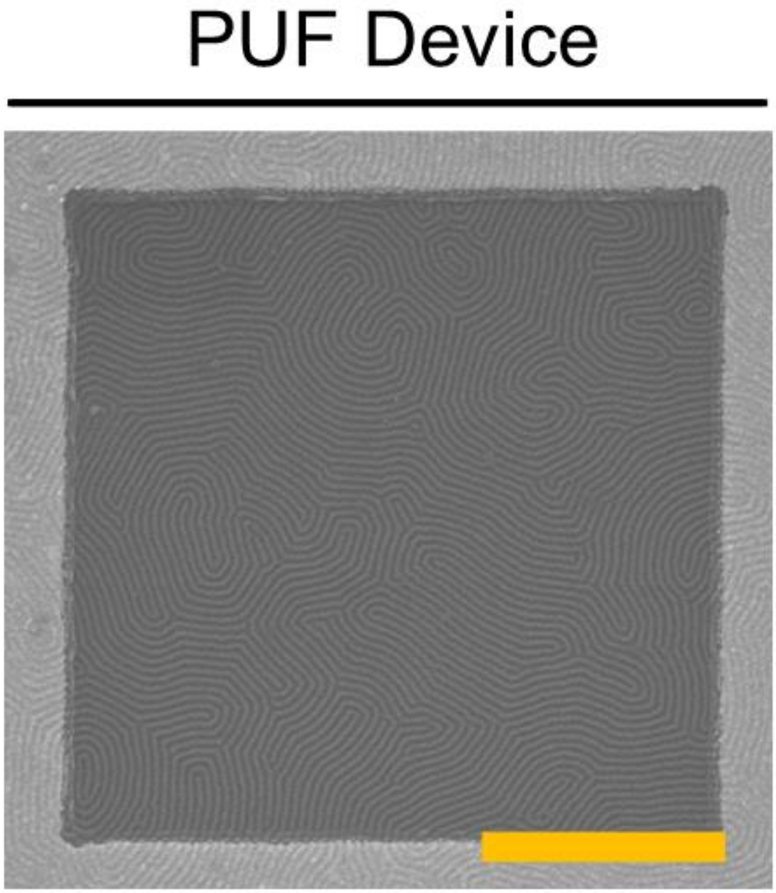

Supplementary Figure S20 | PUF device based on block-copolymer templated SiO_2 substrate. SEM image of a PUF device, where the nanopattern area of interest for authentication/identification is identified thanks to an Au frame. Scale bar is 1 µm.

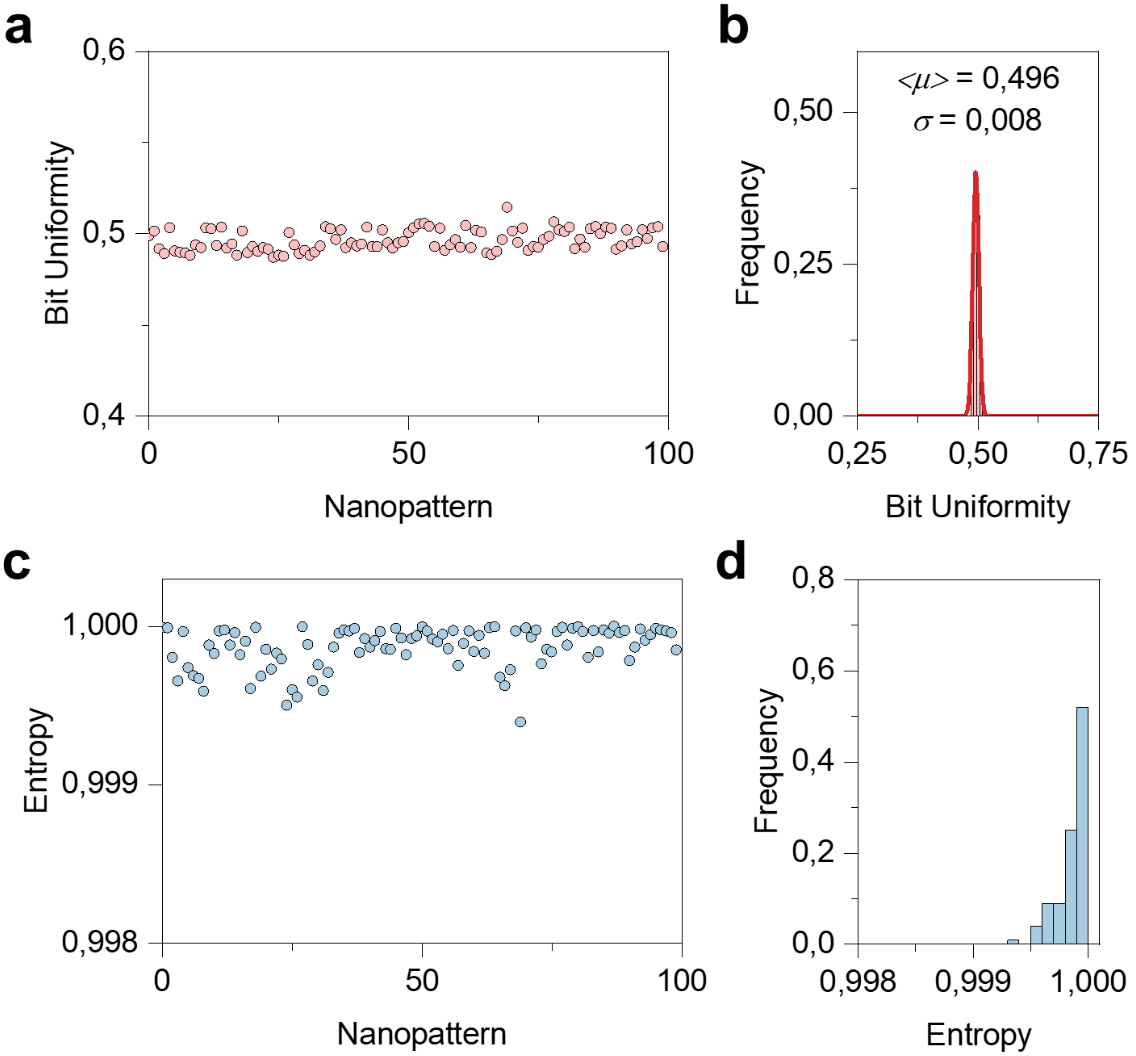

Supplementary Figure S21 | Bit uniformity and unit entropy of binarized lamellar patterns of PUF devices. a. Bit uniformity of the 100 binarized PUF nanopatterns and **b.** corresponding bit uniformity distribution. **c.** Unit entropy of the 100 binarized PUF nanopatterns and **d.** corresponding unit entropy distribution. Note that bit uniformity and unit entropy are here calculated on the whole image, without encoding the pattern into a binary code matrix, by considering images of 100 PUF nanopatterns from the database set of images. A total of 987 x 708 or 996 x 717 pixels is here considered.

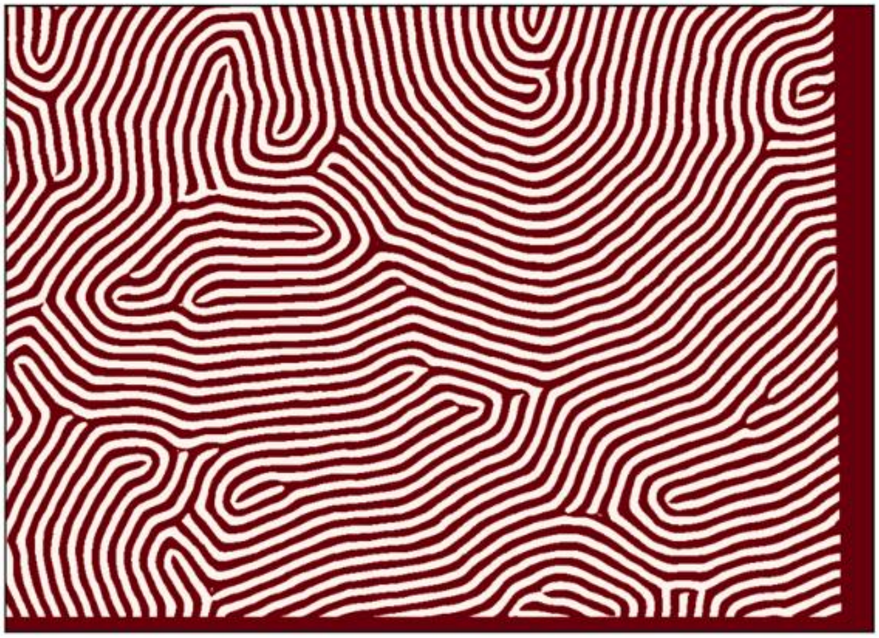

Supplementary Figure S22 | Example of the resulting homographic transformation of a test image when compared with the database image of the same PUF nanopattern. The image represents the homographic transformation of the test image, obtained by comparing the test image with the database image reported in Fig. 4a. As can be observed, the homographic transformation enables the adjustment of the shift/rotation of the acquired test image for a correct overlapping of the test image on the database one.

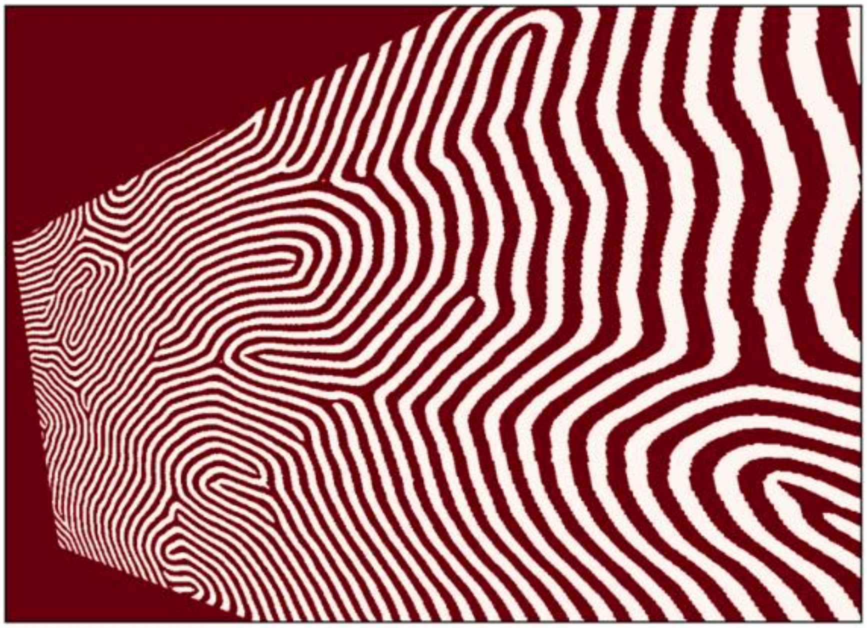

Supplementary Figure S23 | Example of the resulting homographic transformation of a test image when compared with the database image of a different PUF nanopattern. The image represents the homographic transformation of the test image, obtained by comparing the test image with the database image reported in Fig. 4b. The homographic transformation of a test image with a database image of a different nanopattern results in a deformation of the test image leading to an incorrect overlapping of the test image on the database one.

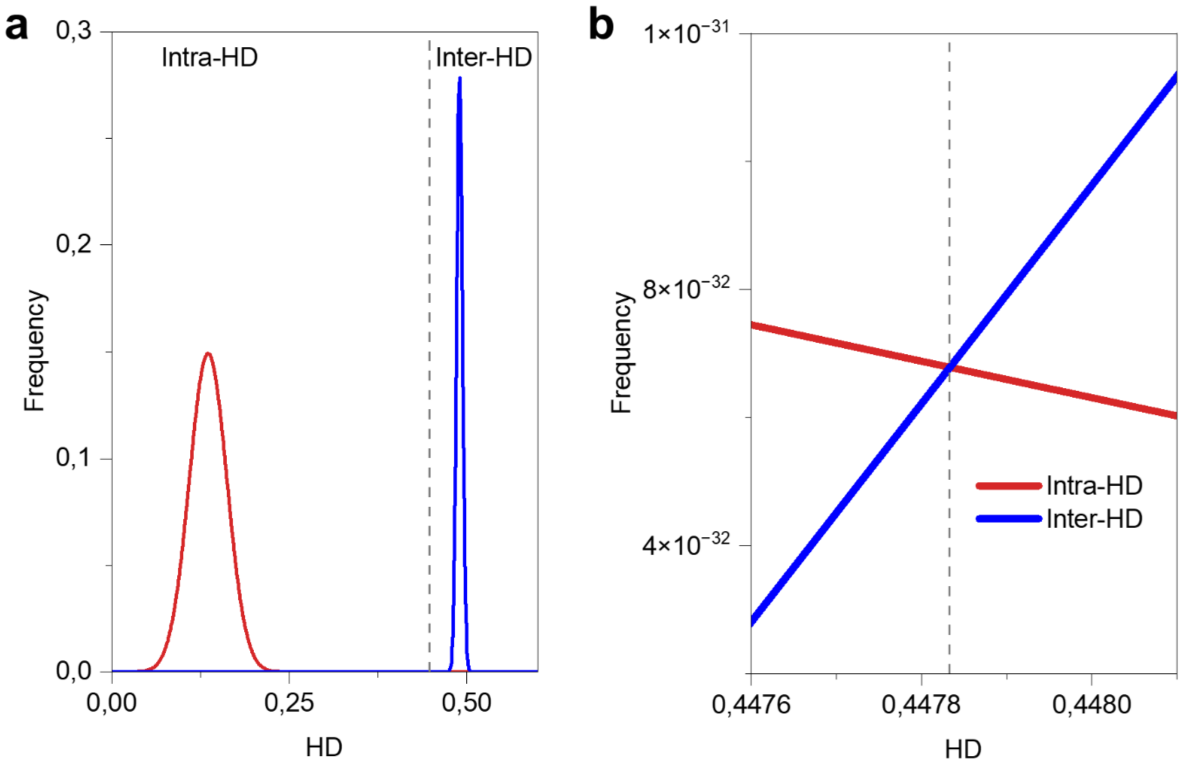

Supplementary Figure S24 | Evaluation of the decision threshold. a. Gaussian distributions of fractional intra-HD and inter-HD obtained by fitting the experimental distribution reported in Fig. 4d, where the test set is compared with the database test, and **b.** detail of the intersection between intra-HD and inter-HD distributions chosen as the decision threshold (the decision threshold is here represented by the dashed line).

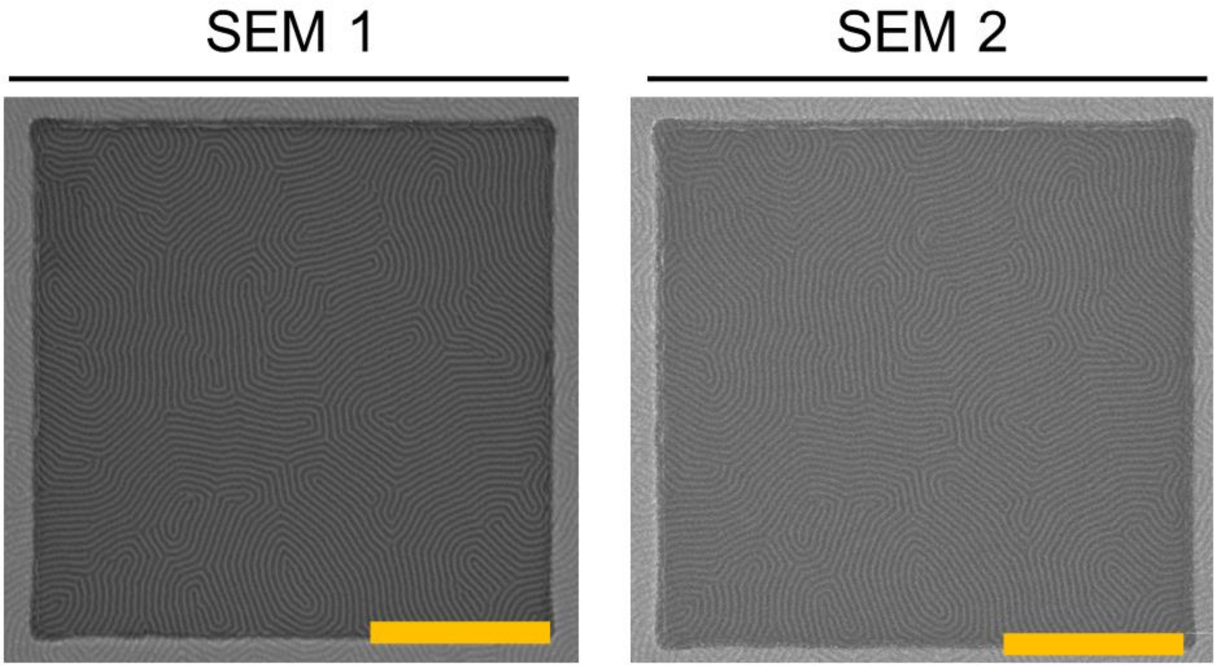

Supplementary Figure S25 | PUF device imaging with different equipment. Example of SEM images of the same nano identifier acquired by two different SEM systems. Images from SEM 1 have been acquired through a FEI Inspect-F, while image SEM 2 have been acquired through FEI Quanta 3D (details in Methods). Scale bars are set to 1µm.

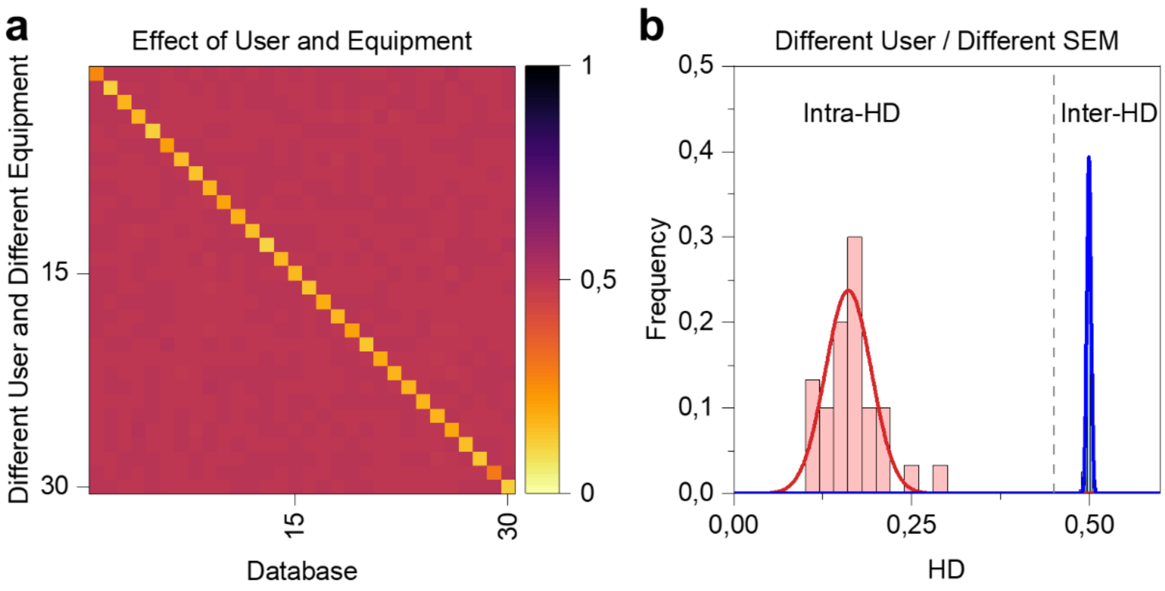

Supplementary Figure S26 | Authentication/Identification through images acquired by a different user operating with a different SEM equipment. a. Heat-map matrix representing the fractional HD between images from a test set of images acquired by a different user operating with a different SEM equipment (SEM FEI Quanta 3D) and images from the database (acquired with a SEM FEI Inspect-F), and **b.** corresponding fractional intra-HD and inter-HD distributions. This test was performed on a subset of 30 PUF patterns.

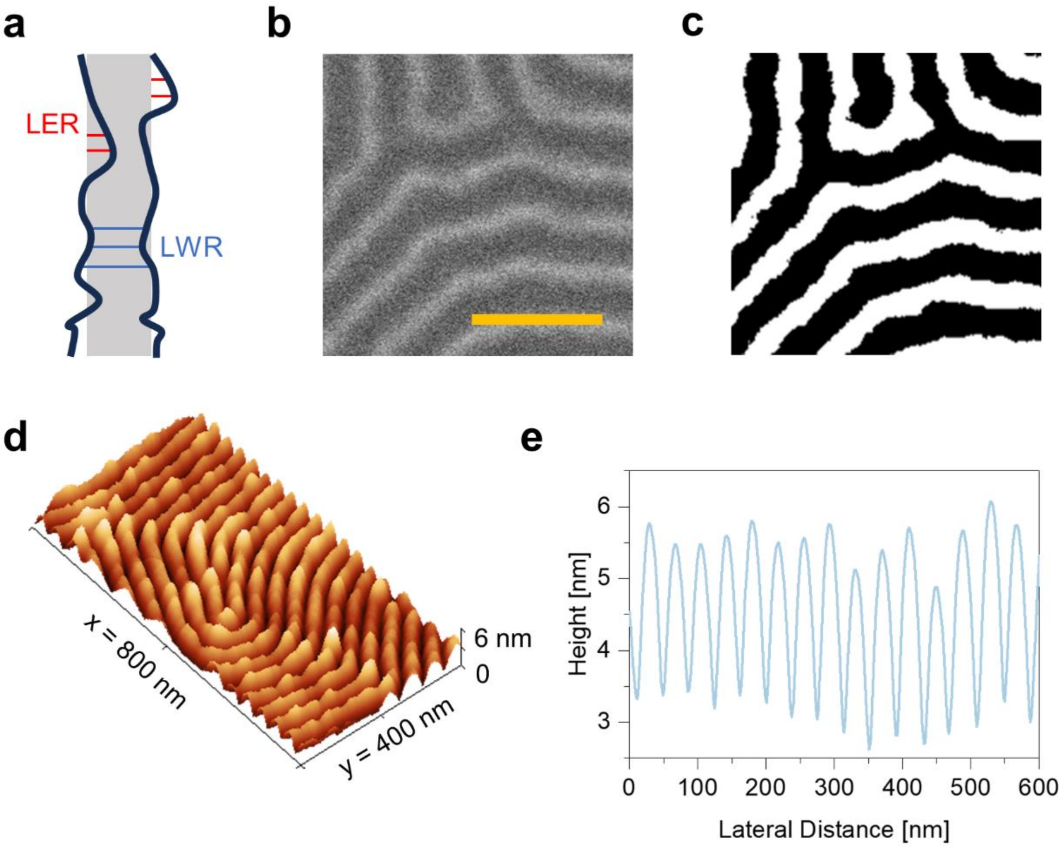

Supplementary Figure S27 | LER, LWR and 3D morphology of the nanopattern. a. Schematics of line edge roughness (LER) and line width roughness (LWR). **b.** SEM image of a fingerprint pattern and **c.** the binarized image by ADA block with Otsu binarization. Scale bar is set to 100 nm. The LER and LWR calculated on binarized image of the engraved nanopattern reported in panel **c.** are 1.39 nm and 2.35 nm respectively. **d.** 3D morphology of a fingerprint pattern measured by AFM in tapping mode over a 400 x 800 nm^2 and **e.** relative height profile.

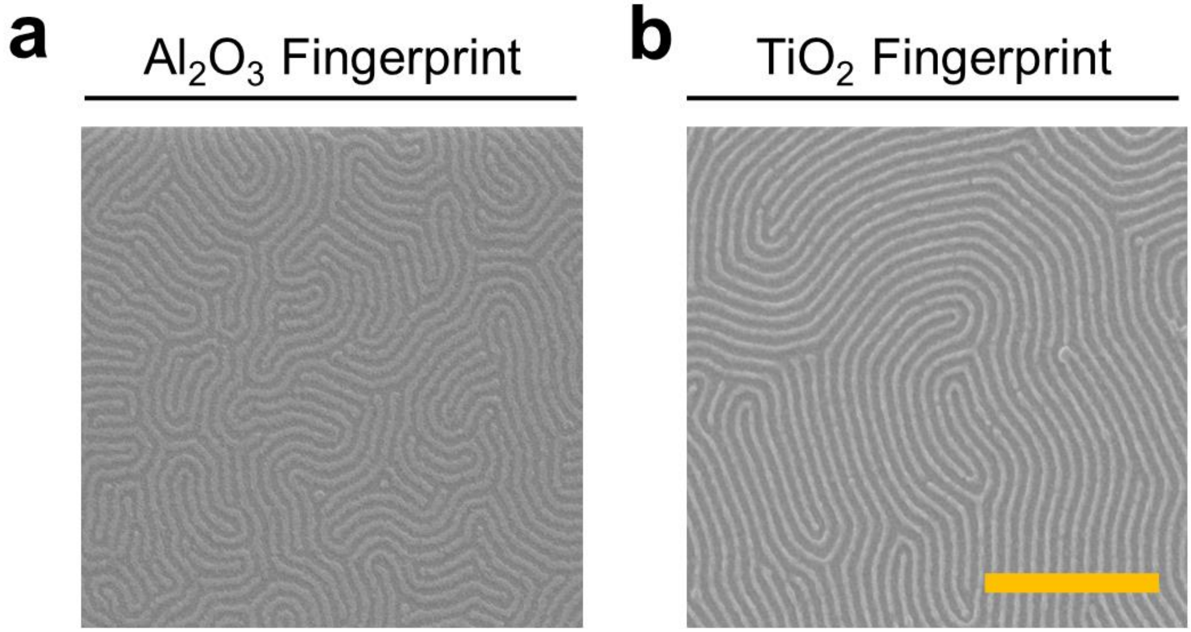

Supplementary Figure S28 | Metal oxide fingerprint patterns | SEM image of fingerprint patterns replicated with **a.** Al_2O_3 and **b.** TiO_2 by means of sequential infiltration synthesis (SIS). Scale bar is set to 500 nm. For this purpose, selective infiltration and growth of metal oxides inside one of the constituent blocks of self-assembled BCPs, is carried out in an atomic layer deposition apparatus. The subsequent polymer removal reveals a nanostructured metal oxide that perfectly replicates the pattern morphology of the self-assembled BCP, which is in this case the fingerprint pattern.

Supplementary Table 1 | Results of NIST SP 800-22 statistical test suite. Results of the NIST SP800-22[1] test performed on the dataset of binary code matrices extracted from 200 nanopatterns, performed by considering binary code matrices with pixel size of 238 × 238 nm^2, i.e., when bit uniformity is ~ 0.5, the HD between binary code matrices of ~ 0.5 and bit entropy is close to 1. Given the proportion results of sequences passing a test (p-value ≥ 0.01), there is evidence that the binary code matrices are random for all the statistical tests applicable. While there is a non-uniform distribution of p-values of binary code matrices for the Frequency (Monobit) Test, for all the other applicable ones the binary code matrices can be considered uniformly distributed. Based on those results, the binary code matrices pass most of the applicable tests of the NIST test suite.

**NIST statistical test[a]**	***p*-value$_T$**	**Proportion α = 0.01 [Pass/Total]**	**Proportion Results[b]**
Frequency (Monobit) Test	0.000014*	198/200	Pass
Frequency Test within a Block	0.616305	197/200	Pass
Cumulative Sums (Cusum) Test	0.062821	197/200	Pass
	0.001824	196/200	Pass
Runs Test	0.181557	198/200	Pass
Approximate Entropy Test	0.002758	198/200	Pass
Serial Test	0.419021	196/200	Pass
	0.989786	198/200	Pass

[a]200 different binary code matrices of 100 bits each were used to perform all possible NIST tests given our data.

[b] The minimum pass rate for each statistical test is approximately = 193 for a sample size = 200 binary sequences.

*Non-uniform distribution of binary code matrices since p-value$_T$ < α, with significance level α = 0.0001.

Supplementary Table 2 | Parameters adopted for the NIST test.

NIST statistical test parameter	Value
Access (n):	100
Bitstreams:	200
Block Frequency Test - block length (M):	20
Approximate Entropy Test - block length (m):	1
Serial Test - block length (m):	4

Supplementary Table 3 | Details on fractional intra and inter-HD mean values and standard deviations evaluated for each authentication/identification test vs the database. Results are obtained from Gaussian fitting of experimental results.

	**Intra-HD**	**Inter-HD**
**Test Set vs Database**	0.14 ± 0.03	0.500 ± 0.003
**Different user/Different SEM** (restricted test set)	0.16 ± 0.04	0.500 ± 0.003
**After 6 Months**	0.15 ± 0.03	0.500 ± 0.003
**After Thermal Treatment at 200 °C for 300 s**	0.16 ± 0.04	0.500 ± 0.003
**After Thermal Treatment at -196 °C for 300 s**	0.17 ± 0.04	0.499 ± 0.003

Supplementary Table 4 | Direct comparison of our work with other existing materials and techniques applied for PUF. The table below reports a comparison of figures-of-merit of various PUF devices, including the dataset size, randomness in terms of unit entropy and/or approximate entropy, uniqueness in terms of Hamming distance, uniformity, area of the normalized bitcell, encoding capacity, aging and temperature stability in comparison to the literature. [a]The randomness of binary-bit sequences were tested by statistical National Institute of Standards and Technology (NIST) test suite (NIST SP 800-22).[1] *Values referred to PUF topography microstructures. [#]Pass rate not specified. [§]Values referred to image-based nanoidentifiers.

Ref	Database set	Approximate Entropy Test (Pass/Total)	Randomness (entropy)	Uniqueness (inter-HD)	Uniformity (bit uniformity)	Normalized bitcell area	Encoding capacity	Aging stability	Temperature stability
Our work[a]	200	198/200	0.99 ± 0.01	0.50 ± 0.06	0.52 ± 0.06	2.38 × 2.38 μm^2	1.3×10^{30}	6 months	-196 – 200 °C / 30 min
Ref[2a]	30	59/60	/	0.495 ± 0.033	0.495	/	2^{227}	Not tested	1000 °C / 1h
Ref[3]	1100	/	/	/	/	/	10^{348}	6 months	-40 – 750 °C
Ref[4]	100	/	/	0.497 ± 0.013*	0.494 ± 0.005*	950 × 950 μm^2	~ 10^{31796}	2 months[§]	Not tested
Ref[5]	90	/	0.9714 ± 0.0416	0.4863 ± 0.0800	51.96 ± 0.19%	204 × 204 μm^2	~ 10^{763}	7 days	200 °C
Ref[6]	15	/	~ 1	~ 0.5	~ 0.5	100 × 100 μm^2	~ 2^{380000}	Not tested	Not tested
Ref[7a]	80	77/80	/	0.4997 ± 0.0363	0.4900 ± 0.0367	/	~ 2^{190}	Not tested	Not tested
Ref[8]	100	/	/	0.5000	0.4996	100 × 100 μm^2	/	Not tested	400 °C / 24h
Ref[9]	9	/	/	49.72 ± 3.37%	50.40 ± 1.67%	0.139 × 0.139 μm^2	/	1.5 months	Not tested
Ref[10a]	30	60/60	Red 60/60<br>Green 60/60<br>Blue 58/60	Red 0.506 ± 0.060<br>Green 0.507 ± 0.066<br>Blue 0.505 ± 0.062	Red 0.499 ± 0.055<br>Green 0.504 ± 0.052<br>Blue 0.507 ± 0.062	/	~ 4^{95}	/	70 °C / 10 days
Ref[11]	300[§]	/	1.080 ± 0.127[§]	0.5007 ± 0.0545[§]	/	0.75 × 0.75 μm^2	2.83×10^{163}[§]	Not tested	150 °C / 1h
Ref[12a]	100	Passed[#]	/	0.38	0.505	10 × 10 mm^2	10^{2500}	11 days	45 °C / 11 days
Ref[13a]	100	86%	/	Ceramic Table Micro<br>0.492 ± 1.8×10^{-5}<br>Ceramic Portable Micro<br>0.484 ± 4.7×10^{-5}<br>Metal Table Micro<br>0.499 ± 1.4×10^{-5}<br>Metal Portable Micro<br>0.494 ± 1.7×10^{-5}	/	/	2^{28735}	/	/
Ref[14]	24	/	~ 1	0.47	~ 1	/	2^{64}	5 days	-175 – 105 °C
Ref[15]	100	/	/	0.494 ± 0.0056	/	200 × 200 μm^2	2^{11848}	Not tested	380 °C / 30 min
Ref[16a]	30	60/60	/	0.5032 ± 0.0458	0.5	7 × 7 mm^2	2^{120}	60 days	Not tested
Ref[17]	15	/	x_{axis} = 0.93 ± 0.06<br>y_{axis} = 0.91 ± 0.05	~ 13	/	640 × 640 μm^2	10^{1233}	Not tested	Not tested
Ref[18]	100	/	~ 1	~ 0.5	/	3.5 × 3.5 mm^2	2^{1750}	Not tested	Not tested

Supplementary Note 1:

BCPs are constituted by two or more different and chemically incompatible homopolymer chains (one hydrophilic and one hydrophobic) that are covalently linked together. Due to the amphiphilic nature of BCPs, even small structural differences of the constituent blocks determine an increase of the free energy resulting in a microphase separation under specific annealing conditions. However, unlike homopolymer blends, the covalent bond linking the two different blocks counterbalances the thermodynamic forces involved in the phase separation. This leads therefore to the in-parallel self-registration of periodic nanostructures in the range of 10 – 100 nm, that is the so-called self-assembly. The main factors that influence the self-assembly process are the polymerization degree (N), which represents the number of monomeric units in a polymer; Flory-Huggins interaction parameter (χ), that describes the excess of free energy of mixing and estimates the immiscibility of the constituent blocks; volume fractions (f_α and f_β) of each homopolymer.[19] Changes in volume fraction affect the morphology and packing symmetry of the resulting phase-separated nanostructures. BCPs with two constituent blocks can self-assemble into four thermodynamically stable phases. Highly asymmetric BCPs with a ratio above 80:20 self-organize in zero-dimensional spheres arranged in a body-centered cubic (BCC) lattice of polymer B embedded in a polymer A matrix. Hexagonally packed (HP) cylinders of the minority component are formed for BCPs with ratio 70:30. Double gyroids are formed for a very narrow compositional ratio interval and two-dimensional lamellae are formed by highly symmetric (ratio 50:50) BCPs.[20] On the other hand, the periodicity (L_0) and the typical dimensions of the resulting nanostructures is determined by the total molecular weight (M_n), given by the multiplication of N with the molecular weight of the monomeric units (M_0) of the BCP.[21]

Supplementary Note 2:

Even if we cannot exclude in principle the possibility of replicating the nanostructure pattern through NIL, this results to be impractical for our application with the state-of-the art of this technology. Replicating defect-free sub-20 nm patterns with NIL technology is challenging due to multiple factors, including the need for flat substrates,[22] ultra-clean environments,[23] and high costs associated with cleanrooms and equipment.[24] These limitations are highlighted also in the International Roadmap for Devices and Systems (IRDS).[25]

Supplementary Note 3:

The localization of the area of interest can be performed by considering markers (not necessarily a frame, but eventually also coordinates with respect to a feature of the object of interest), the realization of a frame with lithograph steps is both cost-effective and time-efficient. In our work, the frames defining the areas of interest were created using electron beam lithography (EBL). Due to the micrometer scale of the frames and the associated "find-me" structures, they can be fabricated quickly using conventional methods such as optical lithography (e.g., 30-second exposure) or direct laser writer (DLW) lithography. The latter process can even be used to create patterns on curved surfaces using a laser beam.[26,27] These types of optical lithography also allow for the use of thick and durable resists (e.g., SU-8) that can pattern rough surfaces. The compatibility between the self-assembly of BCP and lithographically defined structures created via DLW has previously been demonstrated by our group.[28–30]

References

1. Bassham, L. E. *et al. A Statistical Test Suite for Random and Pseudorandom Number Generators for Cryptographic Applications*. https://nvlpubs.nist.gov/nistpubs/Legacy/SP/nistspecialpublication800-22r1a.pdf (2010) doi:10.6028/NIST.SP.800-22r1a.
2. Esidir, A., Pekdemir, S., Kalay, M. & Onses, M. S. Ultradurable Embedded Physically Unclonable Functions. *ACS Appl Mater Interfaces* **16**, 16532–16543 (2024).
3. Sun, N. *et al.* Random fractal-enabled physical unclonable functions with dynamic AI authentication. *Nat Commun* **14**, 2185 (2023).
4. Zhang, J. *et al.* An all-in-one nanoprinting approach for the synthesis of a nanofilm library for unclonable anti-counterfeiting applications. *Nat Nanotechnol* **18**, 1027–1035 (2023).
5. Meijs, Z. C. *et al.* Pixelated Physical Unclonable Functions through Capillarity-Assisted Particle Assembly. *ACS Appl Mater Interfaces* **15**, 53053–53061 (2023).
6. Chen, P. *et al.* Programmable Physical Unclonable Functions Using Randomly Anisotropic Two-Dimensional Flakes. *ACS Nano* **17**, 23989–23997 (2023).
7. Kim, M. S. & Lee, G. J. Visually Hidden, Self-Assembled Porous Polymers for Optical Physically Unclonable Functions. *ACS Appl Mater Interfaces* **15**, 4477–4486 (2023).
8. Zhang, T. *et al.* Multimodal dynamic and unclonable anti-counterfeiting using robust diamond microparticles on heterogeneous substrate. *Nat Commun* **14**, (2023).
9. Porti, M., Redón, M., Muñoz, J., Nafría, M. & Miranda, E. Oxide Breakdown Spot Spatial Patterns as Fingerprints for Optical Physical Unclonable Functions. *IEEE Electron Device Letters* **44**, 1600–1603 (2023).
10. Kiremitler, N. B. *et al.* Tattoo-Like Multi-Color Physically Unclonable Functions. *Adv Opt Mater* **12**, (2024).
11. Kim, J. H. *et al.* Nanoscale physical unclonable function labels based on block copolymer self-assembly. *Nat Electron* **5**, 433–442 (2022).
12. Wu, J. *et al.* A High-Security mutual authentication system based on structural color-based physical unclonable functions labels. *Chemical Engineering Journal* **439**, (2022).
13. Li, Q. *et al.* Intrinsic Random Optical Features of the Electronic Packages as Physical Unclonable Functions for Internet of Things Security. *Adv Photonics Res* **3**, (2022).
14. Dodda, A. *et al.* Graphene-based physically unclonable functions that are reconfigurable and resilient to machine learning attacks. *Nat Electron* **4**, 364–374 (2021).
15. Li, Q. *et al.* Physical Unclonable Anticounterfeiting Electrodes Enabled by Spontaneously Formed Plasmonic Core–Shell Nanoparticles for Traceable Electronics. *Adv Funct Mater* **31**, (2021).
16. Leem, J. W. *et al.* Edible unclonable functions. *Nat Commun* **11**, 328 (2020).
17. Wali, A. *et al.* Biological physically unclonable function. *Commun Phys* **2**, 39 (2019).
18. Nocentini, S., Rührmair, U., Barni, M., Wiersma, D. S. & Riboli, F. All-optical multilevel physical unclonable functions. *Nat Mater* **23**, 369–376 (2024).

19. Bates, F. S. & Fredrickson, G. H. Block Copolymer Thermodynamics: Theory and Experiment. *Annu Rev Phys Chem* **41**, 525–557 (1990).
20. Matsen, M. W. & Schick, M. Stable and unstable phases of a diblock copolymer melt. *Phys Rev Lett* **72**, 2660–2663 (1994).
21. Ferrarese Lupi, F. *et al.* Fine Tuning of Lithographic Masks through Thin Films of PS-b-PMMA with Different Molar Mass by Rapid Thermal Processing. *ACS Appl Mater Interfaces* **6**, 7180–7188 (2014).
22. Ji, R. *et al.* UV enhanced substrate conformal imprint lithography (UV-SCIL) technique for photonic crystals patterning in LED manufacturing. *Microelectron Eng* **87**, 963–967 (2010).
23. Austin, M. D. *et al.* Fabrication of 5nm linewidth and 14nm pitch features by nanoimprint lithography. *Appl Phys Lett* **84**, 5299–5301 (2004).
24. Williams, S. S. *et al.* High-Resolution PFPE-based Molding Techniques for Nanofabrication of High-Pattern Density, Sub-20 nm Features: A Fundamental Materials Approach. *Nano Lett* **10**, 1421–1428 (2010).
25. *INTERNATIONAL ROADMAP FOR DEVICES AND SYSTEMS 2021 UPDATE LITHOGRAPHY THE IRDS IS DEVISED AND INTENDED FOR TECHNOLOGY ASSESSMENT ONLY AND IS WITHOUT REGARD TO ANY*. (2021).
26. Ai, J., Du, Q., Qin, Z., Liu, J. & Zeng, X. Laser direct-writing lithography equipment system for rapid and μm-precision fabrication on curved surfaces with large sag heights. *Opt Express* **26**, 20965–20974 (2018).
27. Zhang, H., Lu, Z. & Li, F. Fabrication of a curved linear grating by using a laser direct writer system. *Opt Commun* **266**, 249–252 (2006).
28. Ferrarese Lupi, F. *et al.* Hierarchical Order in Dewetted Block Copolymer Thin Films on Chemically Patterned Surfaces. *ACS Nano* **12**, 7076–7085 (2018).
29. Murataj, I. *et al.* Hyperbolic Metamaterials via Hierarchical Block Copolymer Nanostructures. *Adv Opt Mater* **9**, 1–9 (2021).
30. Ferrarese Lupi, F. *et al.* Tailored and Guided Dewetting of Block Copolymer/Homopolymer Blends. *Macromolecules* **53**, 7207–7217 (2020).
